# Assessment of Academic Motivation Level of Undergraduate Medical Students of Azad Kashmir, Pakistan

**DOI:** 10.7759/cureus.4296

**Published:** 2019-03-22

**Authors:** Arslaan Javaeed, Ayesha Asghar, Zara Allawat, Quratulain Haider, Khawaja Junaid Mustafa, Sanniya Khan Ghauri

**Affiliations:** 1 Pathology, Poonch Medical College, Rawalakot, PAK; 2 Internal Medicine, Poonch Medical College, Lahore, PAK; 3 Emergency Medicine, Shifa International Hospital, Islamabad, PAK

**Keywords:** ams, medical student, pakistan, azad kashmir, motivation level

## Abstract

Objective

To assess the academic motivation level of undergraduate medical students of Azad Kashmir, Pakistan.

Methods

A total of 378 Poonch Medical College (PMC) students were included in this cross-sectional study. The academic motivation scores of the study subjects were measured using a 28-item, five-point Academic Motivation Scale (AMS) questionnaire originally developed by Deci and Ryan (1985). The tool was checked for internal consistency and was interviewer-administered. Motivation level was quantitatively presented and compared across gender and medical years.

Results

The highest mean motivation score (4.04 ± 2.71) was observed for the statement "Because this will help me make a better choice regarding my career orientation." The following statements showed a statistically significantly higher mean motivation level in females as compared to males: "Because eventually, it will enable me to enter the job market in a field that I like" (p .008) and "for the pleasure that I experience when I read interesting authors" (p .001). But for the statement, "I once had good reasons for going to college; however, now I wonder whether I should continue," males showed a higher motivation level (p. 0.19). A statistically significant difference in mean motivation level was seen across medical years for the following statements: "For the intense feelings I experience when I am communicating my own ideas to others," "For the pleasure that I experience when I read interesting authors," "Because this will help me make a better choice regarding my career orientation," "For the satisfaction I feel when I am in the process of accomplishing difficult academic activities," and "Because I want to show myself that I can succeed in my studies" (p-value <.05).

Conclusion

This study analyzed mean motivation scores for all AMS questions. The study identified that the most common motivational factor for pursuing medical education was because the students thought this will help them make a better choice regarding their career orientation.

## Introduction

Motivation is defined in the literature as follows: "The process whereby goal-directed activity is instigated and sustained [1]; An internal state that arouses, directs, and maintains behavior [2]; An internal force that activates, guides, and maintains behavior over time [3]."

Motivational problems are very common in education. One of the greatest frustrations mentioned by many teachers is that their students are often not motivated to learn. A student that is academically motivated wants to learn, likes learning-related activities, and improves academically [4].

The role of motivation in learning was mentioned through a detailed literature review by Deci and Ryan way back in 1985 [5]. According to these researchers, human behavior is either intrinsically or extrinsically motivated or amotivated [6]. Intrinsic motivation is the drive to pursue an activity simply for the pleasure or satisfaction derived from it, extrinsic motivation is pursuing an activity out of a sense of obligation, or as a means to an end, and amotivation means the absence of intent or drive to pursue an activity due to one’s failure to establish contingencies between the activity and their behavior [5].

After a few years, Vallerand et al. (1989) expanded the initial description, introducing a new division of three subtypes of intrinsic motivation referred to in the academic milieu: orientation toward knowledge (the task is carried out for the pleasure of learning), orientation toward achievement (satisfaction emerges when products are generated or when one’s own limits are overcome), and orientation toward stimulating experiences (it involves activities developed to perceive comforting aesthetics or intellectual or sensorial sensations [6].

Academic Motivation Scale (AMS) is one of the most used instruments to measure the motivation level of students toward learning. Originally, the scale consisted of 28-item seven-point Likert scales [7]. The scale showed a very high level of internal and external consistency in various researches [7-9].

After an extensive literature search through PubMed, Scopus, and Cochrane Library using five keywords (motivation, medical, education, student, and Pakistan), we did not find any study done in Pakistan that measures the academic motivation level of medical students in Pakistan. In the current study, we aim to assess the academic motivation level of undergraduate medical students of Azad Kashmir, Pakistan, using the 28-item Academic Motivation Scale.

## Materials and methods

This cross-sectional study was conducted from April 2018 to October 2018 at Poonch Medical College (PMC), Rawalakot. The sample size was calculated by the following formula: n = z2*p(1-p)/e2. At a prevalence rate of .50, an error rate of .05 and a z value of 1.96, the required sample size was 384. There are 500 undergraduate medical students studying at PMC. In order to get the required sample size, all undergraduate medical students of either gender were recruited in this study. Out of these, 378 returned the questionnaire/agreed/consented to fill the questionnaire (response rate = 75.6%). The study questionnaire used was the 28-item AMS questionnaire [6]. The five-factor variant of the study questionnaire was used here because of its correspondence with the original proposal from Deci and Ryan [5]. The study questionnaire was checked for internal consistency. The study questionnaires, along with informed written consent forms, were distributed to the medical students by the researcher himself. The purpose of the study was clearly explained, and adequate time (30 minutes) was given to each respondent to complete the questionnaire.

Statistical analysis

The Cronbach alpha test was done to assess the internal consistency of the study questionnaire (α=.767). Descriptive statistics were used to present the baseline characteristics. The respondents’ level of motivation was assessed by 28 questions; each of these questions had possible answers arranged in five-point Likert scales. Points in the Likert scale were assigned as follows: 1 = does not correspond at all, 2 = corresponds a little, 3 = corresponds moderately, 4 = corresponds a lot, and 5 = corresponds exactly. These points were used to calculate the mean, standard deviation, skewness, and kurtosis values for the 28 items. The mean motivation score difference between male and female students was calculated by the independent samples t-test. A test for normality was performed using the Kolmogorov-Smirnov and Shapiro-Wilk tests (both tests revealed the non-normal distribution of motivation scores for all 28 items). The mean motivation score difference for different medical years was obtained by the Kruskal-Wallis test.

The analysis was performed at a 95% confidence interval using the Statistical Package for Social Science (SPSS), version 23.0 (IBM, Armonk, NY, USA).

## Results

A total of 378 undergraduate medical students were included in this study and, among them, 267 (70.6%) were females. The frequencies of students from the medical years were as follows: 75 (19.8%) from the first year, 94 (24.9%) from the second year, 63 (16.7%) from the third year, 66 (17.5%) from the fourth year, and 80 (21.2%) from the fifth year. The mean age of all the students was 21.65 ± 1.59 years.

**Figure 1:**
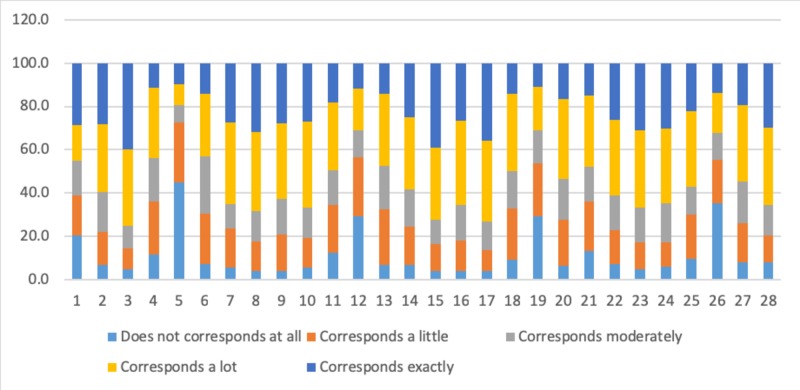
Distribution of all respondents by answers to the 28-item AMS questionnaire AMS: Academic Motivation Scale

**Figure 2:**
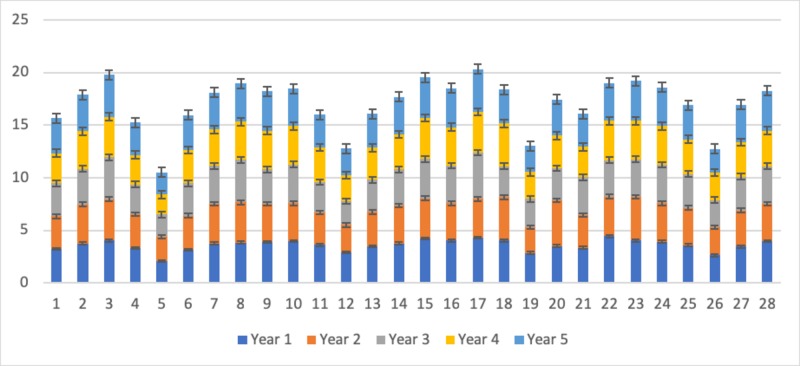
Mean item mean score difference between male and female respondents

**Figure 3:**
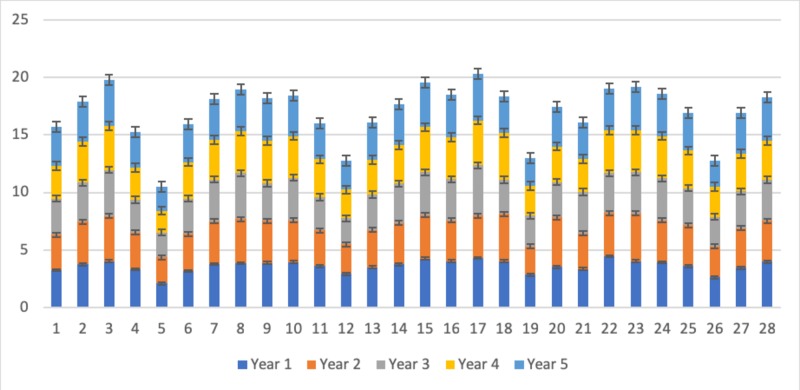
Mean item mean score difference across the undergraduate medical years

The mean motivation scores of all 28 items are presented in Table 1. The highest mean motivation score was observed for item 17 (4.04 ± 2.71) and the lowest score was observed for item 5 (2.11 ± 134). Table 2 shows the responses to the 28-item five-point Likert scale AMS questionnaire. For all the items, except items 1, 5, 12, 19, and 26, the highest number of respondents chose "corresponds a lot." The mean motivation score difference in the 28 items between male and female students is presented in Table 3. The statistically significant differences in the mean motivation levels of males and females were observed for items 10, 11, 12 (p-values .008, .001, and .019, respectively). The mean motivation scores of the students from Years 1 to 5 are presented in Table 4. The statistically significant mean score difference in students depending on the medical years exists for items 4, 11, 17, 20, and 28 (p-values .048, .010, .002, .027, and .016, respectively). Tables 2-4 have been graphically represented in Figures 1-3 in the Appendix.

**Table 1:** Item analysis and descriptive statistics of the study samples

Item No.	Items	Mean	SD	Skewed- ness	Kurtosis
1	Because with only a high-school degree I would not find a high-paying job later.	3.14	1.51	-0.10	-1.45
2	Because I experience pleasure and satisfaction while learning new things.	3.59	1.23	-0.55	-0.74
3	Because I think that a college education will help me better prepare for the career I have chosen.	3.96	1.15	-1.07	0.28
4	For the intense feelings I experience when I am communicating my own ideas to others.	3.07	1.22	-0.15	-1.05
5	Honestly, I don't know; I really feel that I am wasting my time in school.	2.11	1.34	1.03	-0.22
6	For the pleasure I experience while surpassing myself in my studies.	3.20	1.16	-0.12	-0.89
7	To prove to myself that I can complete my college degree.	3.63	1.22	-0.63	-0.71
8	In order to obtain a more prestigious job later.	3.79	1.15	-0.76	-0.38
9	For the pleasure I experience when I discover new things never seen before.	3.66	1.17	-0.55	-0.72
10	Because eventually it will enable me to enter the job market in a field that I like.	3.69	1.17	-0.75	-0.36
11	For the pleasure that I experience when I read interesting authors.	3.20	1.31	-0.23	-1.16
12	I once had good reasons for going to college; however, now I wonder whether I should continue.	2.57	1.39	0.40	-1.18
13	For the pleasure that I experience while I am surpassing myself in one of my personal accomplishments.	3.22	1.17	-0.17	-1.00
14	Because when I succeed in college, I feel important.	3.54	1.34	0.84	8.52
15	Because I want to have "the good life" later.	3.90	1.17	-0.92	-0.15
16	For the pleasure that I experience in broadening my knowledge about subjects which appeal to me.	3.70	1.13	-0.68	-0.41
17	Because this will help me make a better choice regarding my career orientation.	4.04	2.71	14.65	260.61
18	For the pleasure that I experience when I feel completely absorbed by what certain authors have written.	3.70	4.89	9.03	88.14
19	I can't see why I go to college and frankly, I couldn't care less.	2.59	1.37	0.34	-1.20
20	For the satisfaction I feel when I am in the process of accomplishing difficult academic activities.	3.56	3.17	12.03	174.49
21	To show myself that I am an intelligent person.	3.21	1.97	8.28	121.55
22	In order to have a better salary later.	3.81	3.38	11.39	158.59
23	Because my studies allow me to continue to learn about many things that interest me.	3.87	2.42	13.05	223.71
24	Because I believe that a few additional years of education will improve my competence as a worker.	3.72	1.18	-0.73	-0.34
25	For the "high" feeling that I experience while reading about various interesting subjects.	3.39	1.30	-0.42	-1.03
26	I don't know; I can't understand what I am doing in school.	2.55	1.47	0.40	-1.30
27	Because college allows me to experience a personal satisfaction in my quest for excellence in my studies.	3.40	1.21	-0.41	-0.84
28	Because I want to show myself that I can succeed in my studies.	3.66	1.25	-0.74	-0.50

**Table 2:** Distribution of all respondents by answers to the 28-item AMS questionnaire AMS: Academic Motivation Scale

Item No.	Does not correspond at all, N (%)	Corresponds a little, N (%)	Corresponds moderately, N (%)	Corresponds a lot, N (%)	Corresponds exactly, N (%)
1	77 (20.4)	70 (18.5)	61 (16.1)	62 (16.4)	108 (28.6)
2	26 (6.9)	57 (15.1)	70 (18.5)	119 (31.5)	106 (28.0)
3	18 (4.8)	36 (9.5)	40 (10.6)	133 (35.2)	151 (39.9)
4	44 (11.6)	93 (24.6)	76 (20.1)	122 (32.3)	43 (11.4)
5	170 (45.0)	105 (27.8)	30 (7.9)	36 (9.5)	37 (9.8)
6	27 (7.1)	88 (23.3)	100 (26.5)	110 (29.1)	53 (14.0)
7	22 (5.8)	67 (17.7)	43 (11.4)	142 (37.6)	104 (27.5)
8	15 (4.0)	52 (13.8)	52 (13.8)	138 (36.5)	121 (32.0)
9	15 (4.0)	64 (16.9)	62 (16.4)	132 (34.9)	105 (27.8)
10	22 (5.8)	51 (13.5)	52 (13.8)	151 (39.9)	102 (27.0)
11	47 (12.4)	84 (22.2)	61 (16.1)	118 (31.2)	68 (18.0)
12	111 (29.4)	102 (27.0)	48 (12.7)	73 (19.3)	44 (11.6)
13	26 (6.9)	96 (25.4)	77 (20.4)	126 (33.3)	53 (14.0)
14	26 (6.9)	66 (17.5)	65 (17.2)	127 (33.6)	94 (24.9)
15	16 (4.2)	46 (12.2)	43 (11.4)	126 (33.3)	147 (38.9)
16	16 (4.2)	53 (14.0)	61 (16.1)	148 (39.2)	100 (26.5)
17	16 (4.2)	35 (9.3)	51 (13.5)	140 (37.0)	136 (35.9)
18	35 (9.3)	89 (23.5)	65 (17.2)	136 (36.0)	53 (14.1)
19	110 (29.1)	93 (24.6)	58 (15.3)	76 (20.1)	41 (10.8)
20	25 (6.6)	79 (20.9)	72 (19.0)	140 (37.0)	62 (16.5)
21	50 (13.2)	86 (22.8)	61 (16.1)	125 (33.1)	56 (14.9)
22	27 (7.1)	60 (15.9)	60 (15.9)	132 (34.9)	99 (26.3)
23	18 (4.8)	47 (12.4)	60 (15.9)	136 (36.0)	117 (31.0)
24	23 (6.1)	42 (11.1)	68 (18.0)	131 (34.7)	114 (30.2)
25	37 (9.8)	76 (20.1)	50 (13.2)	131 (34.7)	84 (22.2)
26	134 (35.4)	76 (20.1)	47 (12.4)	69 (18.3)	52 (13.8)
27	30 (7.9)	69 (18.3)	73 (19.3)	133 (35.2)	73 (19.3)
28	31 (8.2)	47 (12.4)	53 (14.0)	135 (35.7)	112 (29.6)

**Table 3:** Mean item mean score difference between male and female respondents

Item No.	Male (n = 111) mean ± SD	Female (n = 267) mean ± SD	p-value
1	3.02±1.64	3.19±1.46	.302
2	3.59±1.28	3.59±1.22	.986
3	3.80±1.25	4.03±1.10	.083
4	3.21±1.30	3.01±1.18	.163
5	2.32±1.50	2.03±1.25	.058
6	3.12±1.22	3.23±1.13	.394
7	3.67±1.23	3.62±1.22	.724
8	3.68±1.25	3.83±1.11	.259
9	3.60±1.18	3.68±1.16	.574
10	3.44±1.29	3.79±1.11	.008
11	2.86±1.33	3.34±1.28	.001
12	2.83±1.39	2.46±1.37	.019
13	3.28±1.12	3.20±1.20	.543
14	3.64±1.54	3.51±1.25	.377
15	3.89±1.21	3.91±1.15	.890
16	3.59±1.17	3.74±1.11	.222
17	3.81±1.11	4.13±3.14	.296
18	3.50±5.00	3.78±4.84	.615
19	2.72±1.29	2.54±1.40	.233
20	3.35±1.13	3.64±3.70	.420
21	3.23±3.03	3.19±1.29	.859
22	3.86±5.08	3.79±2.35	.838
23	3.73±1.21	3.92±2.77	.483
24	3.56±1.27	3.78±1.14	.093
25	3.30±1.26	3.43±1.31	.349
26	2.72±1.53	2.48±1.44	.139
27	3.21±1.32	3.48±1.16	.050
28	3.54±1.26	3.71±1.24	.226

**Table 4:** Mean item mean score difference across the undergraduate medical years

Item No.	Year 1 (Mean ± SD)	Year 2 (Mean ± SD)	Year 3 (Mean ± SD)	Year 4 (Mean ± SD)	Year 5 (Mean ± SD)	p-value
1	3.24±1.52	3.09±1.58	3.14±1.51	2.86±1.43	3.35±1.48	.371
2	3.76±1.18	3.70±1.22	3.40±1.28	3.56±1.35	3.46±1.16	.275
3	4.03±1.10	3.93±1.14	3.98±1.08	3.86±1.29	4.00±1.15	.940
4	3.33±1.15	3.20±1.27	2.84±1.15	2.79±1.27	3.09±1.17	.048
5	2.09±1.34	2.29±1.38	2.13±1.34	1.91±1.20	2.09±1.39	.531
6	3.17±1.29	3.22±1.11	3.08±1.13	3.17±1.17	3.30±1.11	.823
7	3.77±1.26	3.74±1.18	3.62±1.11	3.44±1.23	3.54±1.30	.345
8	3.84±1.21	3.83±1.18	4.02±1.05	3.64±1.12	3.64±1.15	.168
9	3.88±0.97	3.65±1.11	3.27±1.31	3.68±1.29	3.74±1.12	.093
10	3.96±1.06	3.62±1.24	3.68±1.19	3.65±1.12	3.55±1.22	.229
11	3.61±1.35	3.09±1.26	2.89±1.25	3.29±1.26	3.13±1.34	.010
12	2.91±1.40	2.60±1.38	2.25±1.41	2.48±1.39	2.54±1.32	.052
13	3.49±1.08	3.26±1.15	3.06±1.13	3.05±1.28	3.20±1.19	.169
14	3.76±1.14	3.60±1.71	3.43±1.21	3.33±1.26	3.55±1.18	.319
15	4.25±0.92	3.79±1.35	3.73±1.15	3.91±1.19	3.85±1.10	.062
16	4.03±1.01	3.55±1.17	3.57±1.15	3.65±1.21	3.69±1.07	.052
17	4.31±0.84	3.68±1.24	4.38±6.21	3.89±1.14	4.05±0.99	.002
18	4.04±5.88	4.11±6.19	2.95±1.11	4.06±6.34	3.20±1.14	.207
19	2.85±1.32	2.44±1.31	2.70±1.52	2.56±1.33	2.46±1.40	.272
20	3.52±1.14	4.33±5.96	3.06±1.12	3.09±1.31	3.45±1.14	.027
21	3.35±1.16	3.10±1.32	3.59±3.84	2.89±1.38	3.16±1.32	.418
22	4.45±6.19	3.76±1.21	3.46±1.22	3.74±4.11	3.60±1.16	.145
23	4.04±1.12	4.14±4.41	3.57±1.25	3.65±1.13	3.79±1.12	.097
24	3.93±1.12	3.64±1.33	3.67±0.98	3.67±1.21	3.69±1.19	.451
25	3.60±1.25	3.55±1.24	3.21±1.36	3.27±1.30	3.26±1.33	.207
26	2.63±1.52	2.66±1.47	2.59±1.49	2.61±1.45	2.26±1.41	.399
27	3.45±1.24	3.44±1.21	3.22±1.21	3.27±1.22	3.54±1.19	.456
28	3.97±1.26	3.56±1.27	3.57±1.17	3.38±1.31	3.79±1.17	.016

## Discussion

Based on the self-determination theory, the AMS probably is the most used instrument for the assessment of student motivation. It has been used in many countries and versions in different languages are available [10]. In the current study, the questionnaire was not translated into Urdu, as all the medical students at PMC had excellent competence in the English language. Research carried out in Spain, the United States of America, Greece, and Paraguay focused mostly on college students and found a better statistic fit for this seven-factor model in contrast with the five-, three-, and one-factor solutions [11-13]. The current study used the five-factor model because of its correspondence with the original Deci and Ryan study and verified internal consistency.

The mean motivation scores of all 28-items were different and ranged from 2.11 to 4.04. The highest mean motivation (4.04 ± 2.71) score was obtained for the statement: "Because this will help me make a better choice regarding my career orientation." This might be due to the study subjects being medical students, as the medical students are, in general, more study- and career-oriented as compared to most of the other disciplines. In 25 out of 28 items, no significant difference in mean motivation score between males and females was observed. This might be because of all the medical students having a similar general ambition, with all of them wanting to be doctors and serving humanity. If the study samples were taken from school- or college-level students, the opposite result could have been observed. The current study observed the fluctuations in the mean motivation levels as the medical year progresses. This study encourages further quantitative and qualitative studies to identify the factors responsible for these fluctuations.

After an extensive literature search, no current study was found that measured the Academic Motivation Level (AML) of medical students. This study measured the AML of Pakistan Medical College students but due to the unavailability of relevant studies, the motivation level could not be compared with similar studies.

Being a single-centered study, the study findings should not be generalized for all Pakistani medical students. Due to the lack of a similar study, the study findings could not be compared with those of other studies. Further similar studies should be conducted to compare the AML of students in different institutions.

## Conclusions

AMS is a well-verified and reliable instrument to measure the motivation levels of students. This study identified the mean motivation level of PMC students and compared the mean differences across gender and medical year. The study identified that the most common motivational factor for pursuing medical education was because the students thought this will help them make a better choice regarding their career orientation. More, similar studies are needed to compare our study findings.
